# Impact of recent COVID-19 infection on liver and kidney transplantation – a worldwide meta-analysis and systematic review

**DOI:** 10.3389/fimmu.2025.1626391

**Published:** 2025-09-29

**Authors:** Nicola Sariye Pollmann, Felix Dondorf, Falk Rauchfuß, Utz Settmacher, Lukas Pollmann, Markus Selzner

**Affiliations:** ^1^ Department of General, Visceral, and Vascular Surgery, Jena University Hospital, Jena, Germany; ^2^ Ajmera Transplant Centre, Toronto General Hospital, University Health Network, Toronto, ON, Canada

**Keywords:** COVID-19, kidney transplantation, liver transplantation, meta-analysis, systematic review, SARS-Cov-2, patient survival, graft survival

## Abstract

**Introduction:**

The shortage of suitable donor organs represents an ongoing global challenge for organ transplantation. During the COVID-19 pandemic, the number of transplantable organs was especially limited. To date, the impact of recent coronavirus-19 (COVID-19) infection on liver and kidney transplant recipients has not been systematically analyzed, which is essential for the development of future transplant management.

**Methods:**

We conducted a systematic review and meta-analysis to assess the clinical outcomes of recent COVID-19 infection in the donor (1) or the recipient (2). A total of 17 studies were considered for systematic review, seven of these were included for meta-analysis.

**Results:**

Transplantation of COVID-19 positive donors did not result in an impaired graft survival for liver or kidney transplantation up to 180-days of follow up. Additionally, a positive COVID-19 donor status was not associated with decreased overall survival in kidney transplant recipients within 180 days of transplantation. Nevertheless, an association was found with decreased overall survival in liver transplant recipients within the 180-day follow-up period.

**Discussion:**

However, the heterogeneity of studies investigating COVID-19 infection of the recipient did not allow a classification of the significance of COVID-19 positive recipients. Conclusively, a COVID-19 positive donor status should not be considered as an exclusive factor for declining a suitable liver or kidney for transplantation.

**Systematic review registration:**

https://www.crd.york.ac.uk/PROSPERO/, identifier CRD42024562551.

## Graphical Abstract

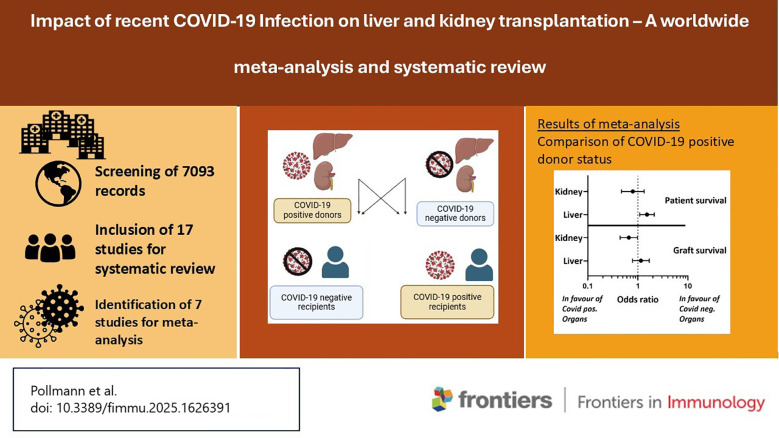

## 1 Introduction

The global impact and management of the coronavirus-19 (COVID-19) pandemic remains an ongoing challenge for transplant society. Until today, a high infection rate of the COVID-19 virus and its variants can be detected in the global population, with over 300–000 confirmed cases and more than 4–500 new deaths reported from September until October 2024 (1). Cumulatively more than 774 million cases and more than seven million deaths were confirmed during the COVID-19 pandemic until November 2024. The analysis of the COVID-19 pandemic is challenging due to the dynamics of the pandemic since the first reported cases in 2020, the worldwide outbreak in 2021 and the development of different viral variants including their predominance in different countries (1). Nevertheless, the COVID-19 pandemic was and remains a worldwide issue, that has not been assessed in detail for liver and kidney transplant recipients to draw lessons for future procedures.

Especially, immunocompromised patients face a higher risk of a severe COVID-19 infection and experience increased morbidity and mortality compared to the general population (2). Therefore, the management of patients with a recent COVID-19 infection represents a current topic in the transplant and allocation process (3).

The reduction of transplant procedures during the past pandemic years translated to a high number of waitlisted patient life-years lost (4). The kidney transplant programs were ranked first for decreased transplant procedures worldwide, followed by lung, liver and heart during the COVID-19 pandemic (4). Especially during the last years of the COVID-19 pandemic, the development and distribution of different vaccines led to a global turning point. Consequently, a decreased infection rate, hospitalization and admission to an intensive care unit was observed in fully vaccinated liver and kidney transplant recipients (5, 6).

Two scenarios of a COVID-19 infection that might impair an upcoming transplant procedure can be defined: the transplantation of a COVID-19 infected donor (1) and the transplantation of a COVID-19 positive recipient (2). For both settings the question remains whether to continue the transplant procedure given the risk of impaired patient or graft survival.

The limitations of international guidance for transplant policies represent an aggravating factor concerning the management of a prior or active COVID-19 infection. Both information and guidance on this topic are limited regarding liver and kidney transplantation.

A recent COVID-19 infection in the recipient is believed to impair patient and graft survival of liver and kidney transplant recipients (7–9). In the context of recent viral or bacterial pneumonia after liver transplantation that occurs after liver transplantation in 15.5% of cases within six days, an association has been identified with prolonged postoperative mechanical ventilation, a longer stay in the intermediate care unit, and a trend toward higher mortality rates (10). Therefore, it is possible that a recipient infected with COVID-19 may experience postoperative pulmonary complications similar to those described. Furthermore, an ongoing COVID-19 infection is associated with a hyperinflammatory response due to cytokine release syndrome, which is linked to acute respiratory distress syndrome and organ dysfunction leading to increased mortality rates and graft impairment in kidney and liver transplant recipients (9).

Furthermore, early statements of the institutions mentioned did not recommend the transplantation of COVID-19 positive donors. As such, the European “Guideline on the quality and safety of organs for transplantation” in the 8th version of 2022 of the “European Directorate for the Quality of Medicines & HealthCare” (EDQM) recommends that organs from COVID-19 positive donors should not be accepted until 14 days after the last symptoms. A clear recommendation to accept organs from COVID-19 positive donors has not been given at this time. Consistent with these recommendations, the acceptance of COVID-19 positive kidney donors was not recommended in 2021 (11). However, for liver transplant patients the “American Association for the Study of Liver Diseases” (AASLD) provided updated recommendations tending toward accepting an incidental COVID-19 positive donor. For COVID-19 positive recipients with active symptoms, a COVID-19 Polymerase Chain Reaction (PCR) test or a computed tomography (CT) scan should be repeated between 14 and 21 days. If symptoms of an active infection are resolved it may be possible to continue with the procedure depending on clinical assessment and the urgency of transplantation (12). Concordantly, the council of transplant infectious diseases recommended that transplant physicians may proceed for non-lung and non-intestine organs with a positive COVID-19 test, if the donor’s death was not COVID-19 related and if the recipient’s consent was obtained (13).

This change in policies and the increasing research on the outcomes of recipients with COVID-19 infection highlights the need for a review of current studies regarding the safety and feasibility of transplants involving infected organs and recipients. Another positive aspect favoring a profound investigation of a recent COVID-19 infection in liver and kidney transplantation is the lack of transmission observed from infected donor organs to recipients (14). While some centers highlight the urgency and need for donor organs and promote the acceptance of COVID-19 positive organs (15), others remain cautious. Therefore, this systemic review and meta-analysis aim to estimate two central questions (1): Can we accept a donor with a recent or ongoing COVID-19 infection and (2) Can we perform transplantation in a recipient with a recent or ongoing COVID-19 infection.

## 2 Materials and methods

A systematic review and meta-analysis ofCOVID-19 infection in (1) donors and (2) recipients of liver and kidney transplants were conducted in accordance with the Preferred Reporting Items for Systematic Reviews and Meta-Analyses (PRISMA) guidelines. The systematic review and meta-analysis were registered in the PROSPERO database (Registration-number CRD42021269372).

### 2.1 Search strategy

The PubMed, Medline, Cochrane, Europe PubMed Central (PMC) and World health organization (WHO) COVID-19 databases were searched from January 2024 to January 2025 by two independent reviewers. Articles published from January 2020 until January 2025 were screened for eligibility. A combination of the following Medical Subject Heading (MeSH) terms was used to identify articles investigating a recent COVID-19 infection in liver and kidney transplant recipients:

- “coronavirus,” “SarsCov,” “SarsCov2,” “SARS-Cov-2,” “Severe Acute Respiratory Syndrome,” “COVID,” “COVID-19,” “HCoV-19”, “Coronavirus infection”
- ”liver”, “hepatic”, “kidney”, “renal”
- ”transplantation”, “transplant”, “graft”

The detailed search string for each of the databases is provided in the **Supplementary Table 3**. All records were transferred to a reference list and subsequently screened for duplicates by two independent authors. Titles and abstracts of each predefined reference list were screened and assessed for eligibility by two independent authors. Any disagreement was resolved by means of point-by-point discussion.

### 2.2 Eligibility criteria

Donors and recipients were classified as infected with COVID-19 if they had a positive COVID-19 PCR test or typical findings on preoperative CT scan along with clinical signs of COVID-19 infection at the time of transplantation or with a very close time to transplantation (less than 30 days before transplantation). Studies investigating transplantation of kidney and liver grafts were included. Reported results and studies of a COVID-19 infection in heart, lung, pancreas, intestine transplantation and multi-organ transplantation were excluded. Furthermore, studies with less than five patients or with pediatric patients (less than 18 years old), as well as reviews and letters to the editor were excluded. All non-English, investigational, animal and *in vitro* were excluded. Conclusively, only reports written in English language were included in the investigation. Furthermore, all studies without a control group were excluded from meta-analysis.

### 2.3 Data extraction for systematic review

Systematic data acquisition using predefined criteria was conducted and corresponding authors of records that reported unspecific data that may lead to misinterpretations were contacted for clarification. Baseline characteristics of the records were extracted: publication author and date, the organ of interest (liver, kidney), the source of data (single center data, multicenter database or national database). The infection status of the donor and the recipient was accessed, and the studies were searched for the presence of a control group. Furthermore, the type of COVID-19 tests and the median time of the COVID-19 infection prior or after the solid organ transplantation were determined. Next, the donor and recipient characteristics were summarized, including age, body mass index (BMI) and gender. This was followed by calculation of a weighted mean, based on the size of the study population. For the donors, the proportion of donation-after cardiac death (DCD) was retrieved.

### 2.4 Meta-analysis

Studies reporting outcomes after a recent COVID-19 infection in the donor were selected for meta-analysis. The primary outcome parameter for meta-analysis was the recipient survival 30-, 90- and 180 days after transplantation. The secondary outcome parameter was survival of the graft 30-, 90- and 180 days after transplantation.

#### 2.4.1 Risk of bias assessment

The Risk of Bias in the selected studies for meta-analysis was compared according to the Robinson-I questionnaire for non-randomized studies and visualized using the RobVis-tool (16, 17).

#### 2.4.2 Data analysis and visualization

Recipient and graft survival after transplantation of a COVID-19 positive donor was compared with recipient and graft survival after transplantation of a non-COVID-19 positive donor. Analysis and visualization were performed using the online platform Cochrane Review Manager (Review Manager (RevMan), The Cochrane Collaboration, available at revman.cochrane.org). The Mantel-Haenszel statistical method with fixed effects was selected to calculate the odds ratio and 95% confidence interval.

## 3 Results

**Figure 1:**
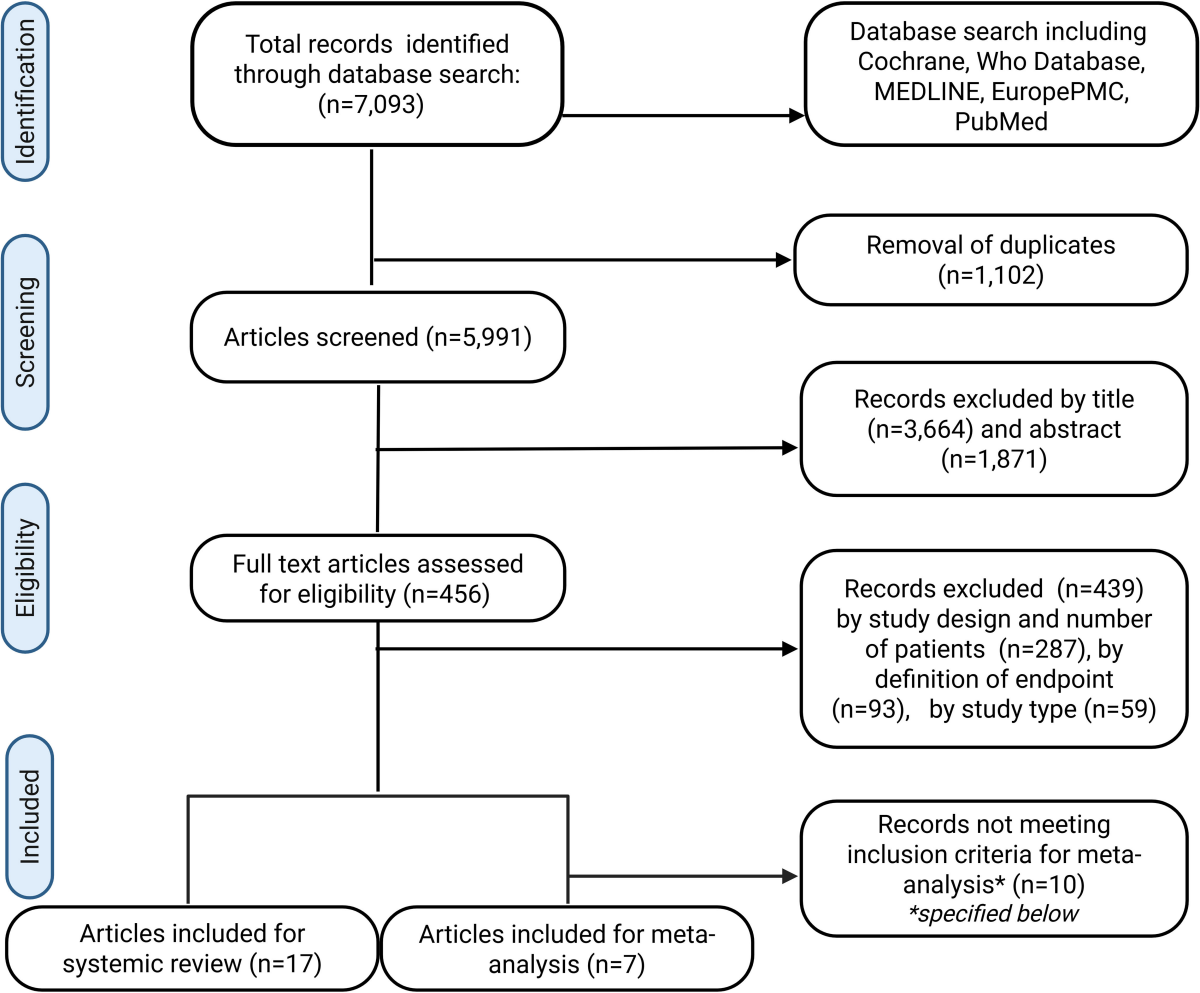
PRISMA-Diagram for the systematic literature search of the Pubmed, Medline, Cochrane, Europe PMC and WHO COVID-19 databases. Inclusion criteria for meta-analysis: Donor-related studies with a COVID-19 negative control group, patient and graft survival reported at 30, 90 and or 180 days after transplant.

A total number of 7093 records were identified by MeSH term search of the mentioned databases. Following the elimination of 1102 duplicates, 5991 records were examined by title and abstract. A total of 465 articles were assessed for eligibility by full-text screening. Following a comprehensive evaluation of the literature, 17 studies were included in the systematic review (see **Figure 1**). A total of 15 of the 17 studies reported a COVID-19 infection of the donor and two studies reported a recent COVID-19 infection of the recipient. A total of seven studies were included in the meta-analysis. These studies investigated the impact of a donor COVID-19 infection on the postoperative outcome of the corresponding recipients. Due to the substantial variability among recipient-related studies, these were included in the systematic review but not in the meta-analysis.

### 3.1 Systematic review

#### 3.1.1 COVID-19 infection in donors

**Figure 2:**
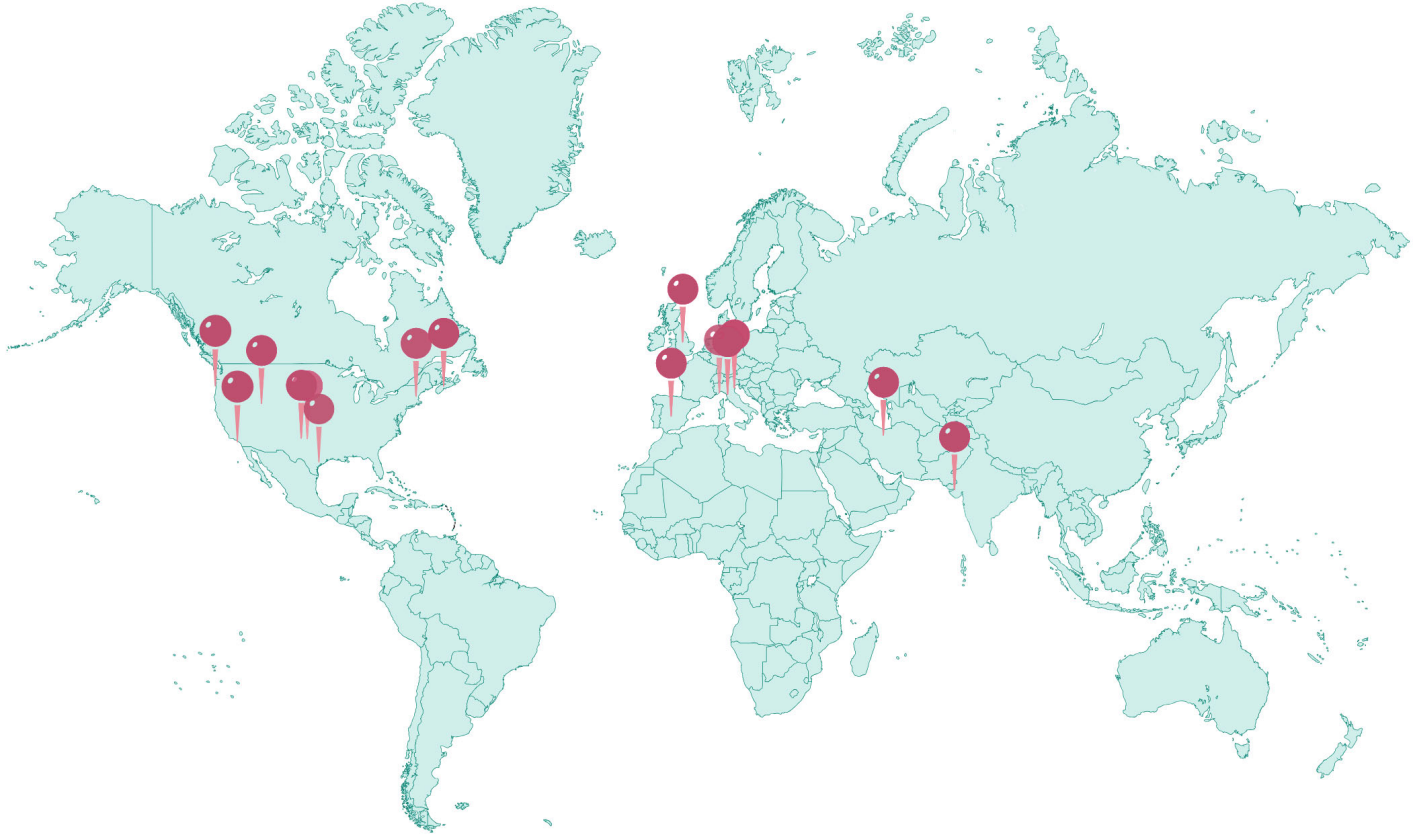
Publication countries.

The outcome of recipients following a liver or kidney transplant from a COVID-19-positive donor was examined in 15 studies. Ten of these studies included a control group with recipients from COVID-19-negative donors. Studies analyzing a recent COVID-19 donor infection reported both single and multicenter experiences. Accordingly, the number of recipients who received a transplant from a COVID-19 positive donor ranged from five to 1533. A total of eight studies reported on single center experiences regarding kidney or liver transplants from COVID-19-positive donors. Moreover, seven multicenter studies were conducted. Among these, five utilized data from the United States’ “Organ Procurement and Transplantation Network” (OPTN) database (18–22) (**Figure 2**). In addition, multicenter studies were conducted in two European countries: Spain and Italy (23, 24) (**Figure 2**). The time interval between the donor’s first positive COVID-19 tests, and transplantation varied across studies, ranging from 24 days to one day prior to transplantation, as shown in **Table 1**.

**Table 1:** Baseline characteristics of included studies for systematic review.

Author (year)	Organs	Recipients donor cov. + (n)	Recipients donor cov. - (n)	Country	Database	Control group
Donor-related studies						
Bock et al. (2022) (18)	kidney, liver, other ^1^	269	44500	USA	OPTN	Yes
Gold- man et al. (2022) (20)	kidney, liver, other ^2^	1241	21946	USA	OPTN	Yes
Koval et al. , (2022) (28)	kidney	115	105	USA	SC	Yes
Sanchez-Vivaldi et al. (2022)	kidney	9	–	USA	SC	No
Schold et al. (2022) (21)	kidney, liver, heart	284	16591	USA	OPTN	Yes
Koval et al. ^2^, (2022) (28)	kidney	10	–	USA	SC	Yes
Ali et al. (2021)	kidney	5	–	UK	SC	No
Villalonga et al. (2022) (24)	kidney, liver, heart	69	–	Spain	MCN	No
Dhand et al. (2022) (19)	kidney, liver, other ^3^	193	18429	USA	OPTN	Yes
Meng-meng et al. (2023)	kidney	1533	50656	USA	OPTN	Yes
Yamauchi et al. (2023) (29)	kidney	61	183	USA	SC	Yes
Grossi et al. (2022)	liver	10	–	Italy	SC	No
Roma-gnoli et al. (2021) (23)	liver	10	–	Italy	MCN	No
Martini et al. (2023)	liver	25	258	Italy	SC	Yes
Connor et al. (2023) (27)	liver	29	472	USA	SC	Yes
Recipient-related studies						
Meshram et al. (2021) (25)	kidney	22	NA	India	MCN	Yes
Moradi et al. (2023) (26)	liver	48	566	Iran	SC	Yes

Table legends: other^1^: pancreas, lung, heart, other^2^: pancreas, lung, heart, intestine; other^3^: heart, pancreas, SC, single center; MC, multi-center; MCN, multi-center nationwide.

Baseline characteristics of liver and kidney donors within the aforementioned studies were compared regarding age, gender and BMI and the proportion of DCD (**Table 2**). The results indicated a higher percentage of males among COVID-19 positive donors compared to those without infection. Accordingly, the proportion of male COVID-19 positive liver donors 68.1% (n = 764), compared with 62.8% (n = 27855) of male donors among those who tested negative for the virus. In contrast, the age of donors included in this analysis was found to be comparable between those tested positive and negative for COVID-19, as shown by the mean age of COVID-19-positive kidney donors (38.8 years, interquartile range [IQR]: 28–50.8), and the mean age of kidney donors who tested negative for the virus (40.3 years, IQR: 28.3–52.7). In addition, the BMI of kidney and liver donors was comparable in those with and without COVID-19 infection.

**Table 2:** Donor characteristics.

Variable	Donor cov. +	Donor cov. -
Liver transplantation	n = 1123	n = 44354
Age (mean, IQR) in years	35.8 (26.5 – 47)	38.8 (27.3 – 51)
BMI (mean, IQR) in kg/m²	28.6 (24.3 – 33.5)	27.4 (23.5 – 32.4)
Gender (% male)	68.1 (n = 764)	62.8 (n = 27855)
DCD organ (%)	31.5 (n = 354)	21.6 (n = 9581)
Kidney transplantation	n = 1146	n = 43873
Age (mean, IQR) in years	38.8 (28 – 50.8)	40.3 (28.3 – 52.7)
BMI (mean, IQR) in kg/m²	28.6 (24.3 – 33.5)	27.4 (23.5 – 32.4)
Gender (% male)	68.6 (n = 782)	63.1 (n = 27684)
DCD organ (%)	35.4 (n = 406)	25.3 (n = 11100)

IQR, Interquartile range.

Moreover, a higher percentage of DCD was observed in the group of COVID-19 positive donors. As such, the percentage of DCD donations among COVID-19 positive liver donors was 31.5% (n = 354) versus 21.6% (n = 9581) in COVID-19-negative liver donors. In a similar vein, a comparative analysis of kidney transplantation data reveals that the proportion of DCD donation among COVID-19 positive donors was 35.4% (n = 406) versus 25.3% (n = 11100) in COVID-19 negative donors. All donor characteristics are shown in **Table 2**.

An analysis of the baseline characteristics of COVID-19-negative recipients of liver transplants from a COVID-19 positive donor showed a tendency toward a higher age in recipients who received a liver from a COVID-19 positive donor compared to those who received a liver from a COVID-19 negative donor (58 years [IQR: 47 – 65.3] to 56 years [IQR: 44.7 – 64], respectively). Conversely, in kidney transplantation, COVID-19 negative recipients from COVID-19 positive donors were younger with a mean age of 47.7 years (IQR: 43.3 – 63) compared to COVID-19 negative recipients from COVID-19 negative donors (54.4 years [IQR: 42.5 – 63.5]). Furthermore, there were no major differences in gender distribution between liver and kidney recipients, who were not infected and both COVID-19 negative and COVID-19 donors. COVID-19-negative liver recipients from COVID-19-negative donors demonstrated a tendency toward a higher mean Model for End-Stage Liver Disease (MELD) with 30 (IQR: 21 – 37) compared to liver recipients of COVID-19-positive donors (27 [IQR:19 – 35]). All recipient characteristics are shown in **Table 3**.

**Table 3:** Recipient characteristics.

Variable	Recipient donor cov. +	Recipient donor cov. -
Liver transplantation	n = 682	n = 33689
Age (mean, IQR) in years	58 (47 – 65.3)	56 (44.7 – 64)
BMI (mean, IQR) in kg/m²	29.7 (27.8 – 35.2)	28.9 (24.8 – 33.9)
Gender (% male)	58.8	62.2
MELD (mean, IQR)	27 (19 – 35)	30 (21 – 37)
Kidney transplantation	n = 1308	n = 52103
Age (mean, IQR) in years	47.7 (43.3 – 63)	54.5 (42.5 – 63.5)
BMI (mean, IQR) in kg/m²	29.9 (24.9 – 34)	–
Gender (% male)	64.5	61.45

IQR, Interquartile range.

#### 3.1.2 COVID-19 infection in recipients

After reviewing all records of recipients with a positive COVID-19 status within 30 days of transplantation, only two studies met the inclusion criteria, see **Table 1**. A multicenter study was conducted in India to compare the outcomes of kidney transplant recipients with early postoperative COVID-19 infection with two other patient cohorts (1): patients on the waiting list who had COVID-19 infection and (2) kidney transplant recipients who tested negative for COVID-19 (25). In this study, a total of 22 kidney transplant recipients with an early COVID-19 infection out of 838 recipients were examined between March 2020 and May 2021. The mortality rate was the highest among patients on the waiting list who tested positive for COVID-19 (15.5%). Kidney transplant recipients who were recently infected with COVID-19 exhibited a lower mortality rate compared to recipients who tested negative (4.5% and 8.5%, respectively) (25).It is noteworthy that none of the recipients had been vaccinated prior to transplantation. The second study was carried out at a single center in Iran and included 48 liver transplant recipients with early postoperative COVID-19 infection. The authors compared the mortality of liver transplant recipients with early postoperative COVID-19 infection with that of 566 recipients who tested negative for the virus (26). A subgroup analysis of acute versus elective liver transplantation was performed. In elective liver transplants, a higher mortality was observed in COVID-19-positive recipients (47.1%) compared to COVID-19 negative recipients (24.1%). Whereas in acute transplants the mortality of recipients with and without early postoperative COVID-19 infection were similar (42.9% versus 46.4% respectively) (26). The authors indicated that the majority of patients included received a vaccination against COVID-19. However, they did not provide detailed information regarding the number of patients who received the vaccine. Both studies, reporting on early postoperative COVID-19 infection of transplant recipients, are limited due to the small number of patients included for analysis.

### 3.2 Meta-analysis

#### 3.2.1 Study characteristics and risk of bias

For meta-analysis, seven studies were identified. These reported on overall recipient and graft survival after transplantation of COVID-19 negative and positive donors. Of these, two studies reported experiences from a single center (27, 28), and five studies were multicentric. A total of four multicenter studies analyzed the OPTN database (18–21).

Three studies examined the early pandemic period from 2020 to 2021, while two additional studies included patients from 2021 to 2022 (18–20, 28). One study included patients from 2020 to 2022, while another study included patients from 2016 to 2022 (27, 29). Consequently, different variants of the virus dominated during the periods analyzed. However, the predominant variants of the virus were only assessed by Koval et al. (28). Furthermore, the proportion of vaccinated recipients varied across the studies that were incorporated into the meta-analysis. While Connor et al. and Yamauchi et al. highlighted that all recipients were vaccinated, Koval et al. reported a vaccination rate of 88% among recipients of COVID-19 negative donors and 93.2% among recipients of COVID-19 positive donor (27–29). However, the four studies that employed the OPTN database did not provide any information regarding the vaccination status of the patients included (18–21). Only Yamauchi et al. provided information about the donor vaccination status and an antiviral treatment of COVID-19 infected donors (29). In addition, the two studies conducted by Yamauchi et al. and Connor et al. described the proportion of postoperative antiviral prophylaxis of the recipient after organ transplantation from a COVID-19 positive donor (27, 29).

**Figure 3:**
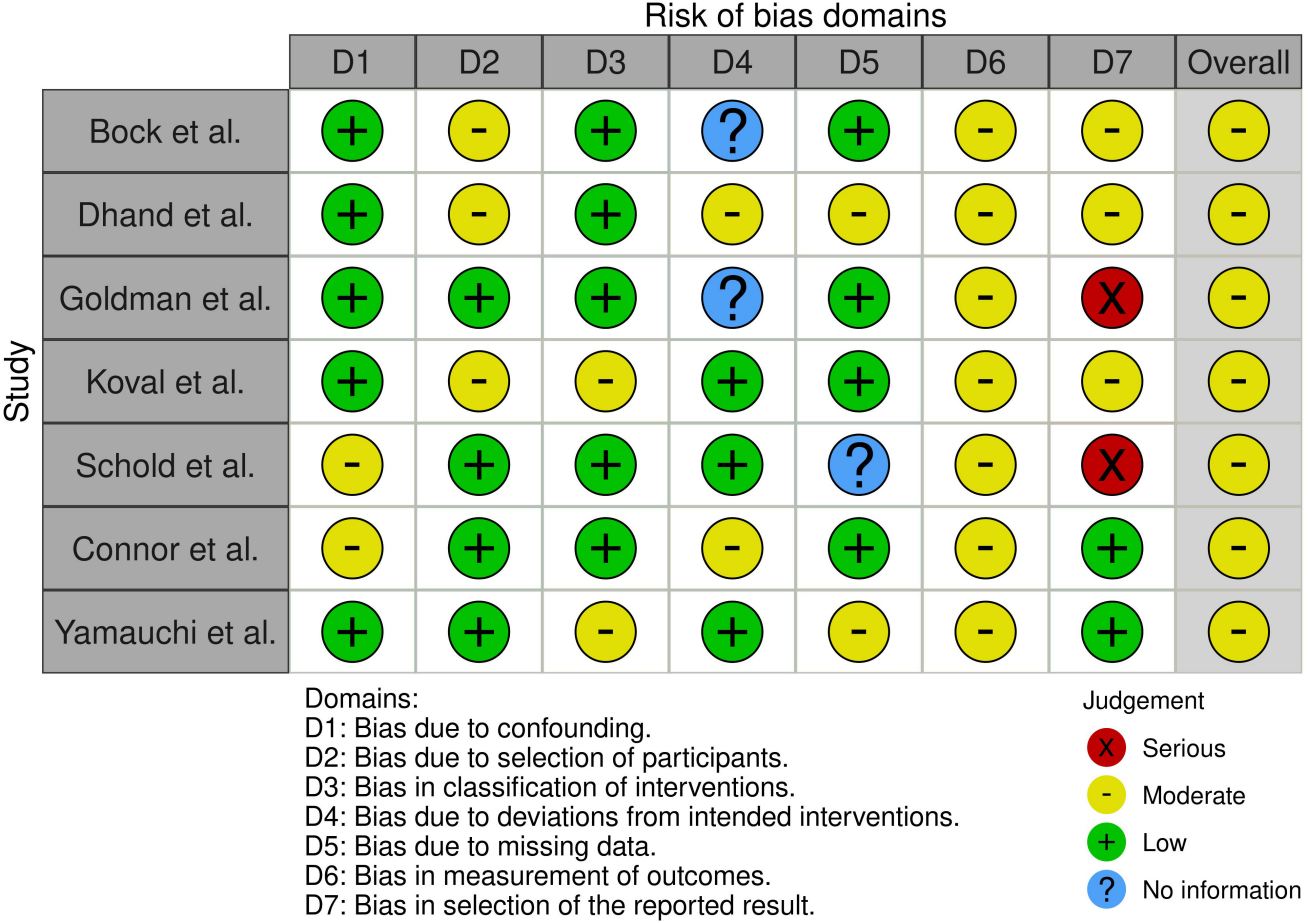
Risk of bias of the included studies utilizing the Robinson-I questionnaire and the RobVis tool.

Consequently, the outcomes regarding graft and recipient survival of the included studies may have been affected by factors such as vaccination of the donor and recipient, antiviral treatment of the donor, and postoperative antiviral prophylaxis. Additionally, the assessment of outcome parameters indicated moderate bias, attributable to the heterogeneity of the reported patient- and graft-related outcomes. The multipart analysis of bias demonstrated that all studies incorporated into the meta-analysis exhibited low to moderate bias with respect to confounding, selection of participants, classification of or deviation in interventions, and integrity of the data set. Nonetheless, the analysis of bias in outcomes and reported results was found to be moderate. Consequently, the overall risk of bias in outcome measurement was assessed as moderate in all analyzed studies (**Figure 3**).

#### 3.2.2 Patient and graft survival

**Figure 4:**
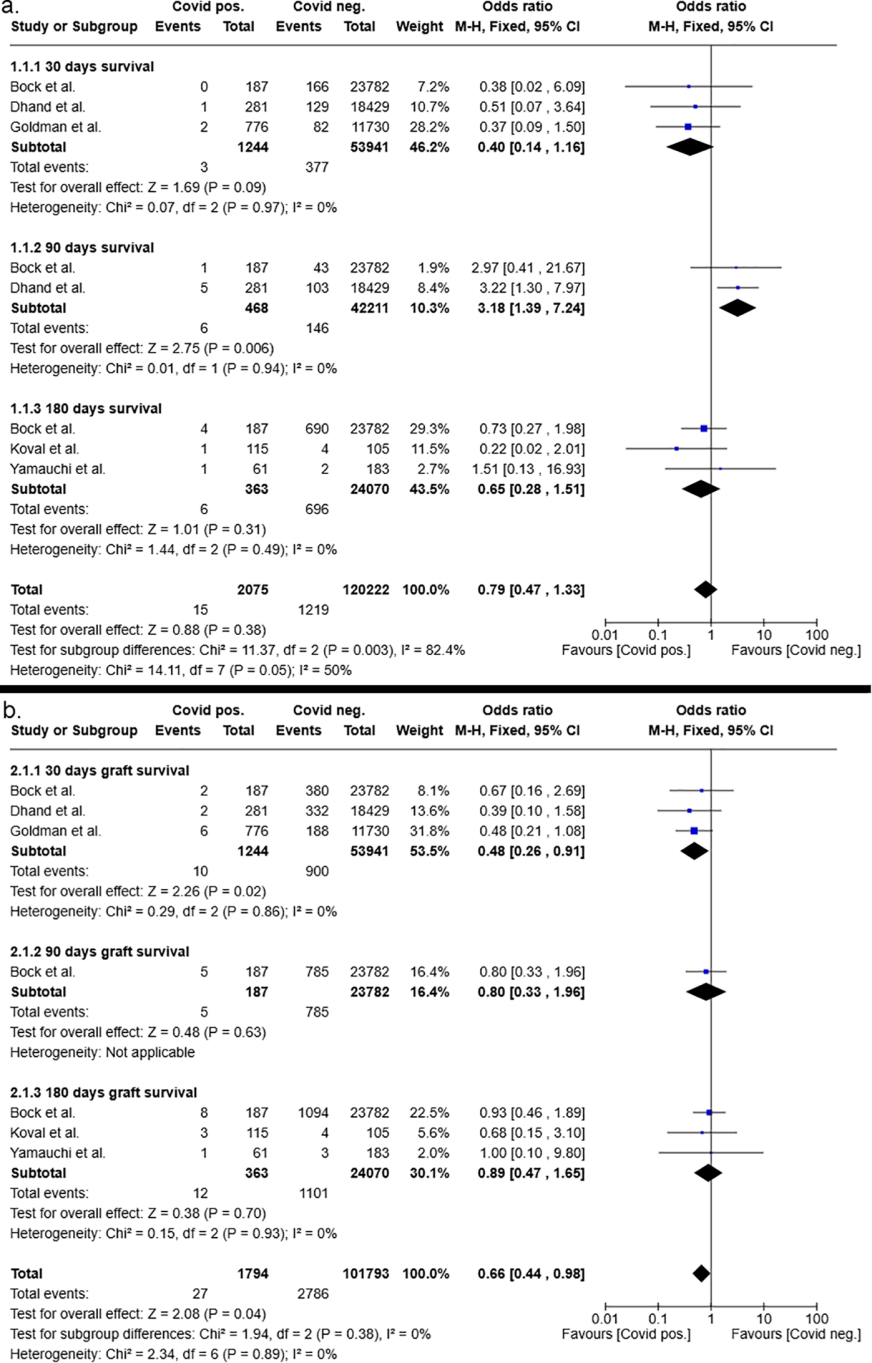
Postoperative 30-, 90- and 180-days patient **(a)** and graft **(b)** survival after kidney transplantation from COVID-19 positive and COVID-19 negative donors.

The survival rates of patients and grafts after liver and kidney transplants from COVID-19 negative and COVID-19 positive donors were determined 30, 90, and 180 days after transplantation. Subsequently, the overall odds ratios and odds ratios at different time points for patient and graft survival were determined. The analysis of patient survival revealed that kidney transplant recipients who received a graft from a COVID-19 positive donor showed a tendency toward a higher survival rate compared to those with COVID-19 negative donors (OR: 0.79, [95% CI 0.47–1.33], p=0.38 (**Figure 4**). Consequently, the 30- and 180-day survival rates of patients following kidney transplantation exhibited odds ratios in favor of kidneys from COVID-19-positive donors with 0.40 (95% CI 0.14–1.16, p=0.09) and 0.65 (95% CI 0.28–1.51, p=0.31), respectively. In contrast, 90 days after kidney transplantation, recipients with COVID-19 negative donors had a significantly higher survival rate compared to those of COVID-19 positive donors (p=0.006). Nonetheless, the calculated odds ratio at 90 days following kidney transplantation exhibited a broad 95% confidence interval with a range from 1.39 to 7.24 (OR: 3.18, p=0.006).

Consistent with patient survival, the analysis of overall graft survival after kidney transplantation showed a significantly higher graft survival rate for recipients of COVID-19 positive donors compared to those kidney recipients who received a graft from a negative donor (OR:0.66 [95%CI 0.44 – 0.98], p=0.04). At the individual time points of 30, 90 and 180 days after transplantation, both odds ratios and 95% confidence intervals demonstrated results that were indicative of an improved graft survival in the patient cohort who received a graft from a COVID-19 positive donor compared to recipients with negative donors. However, the results were not significant (**Figure 4**).

**Figure 5:**
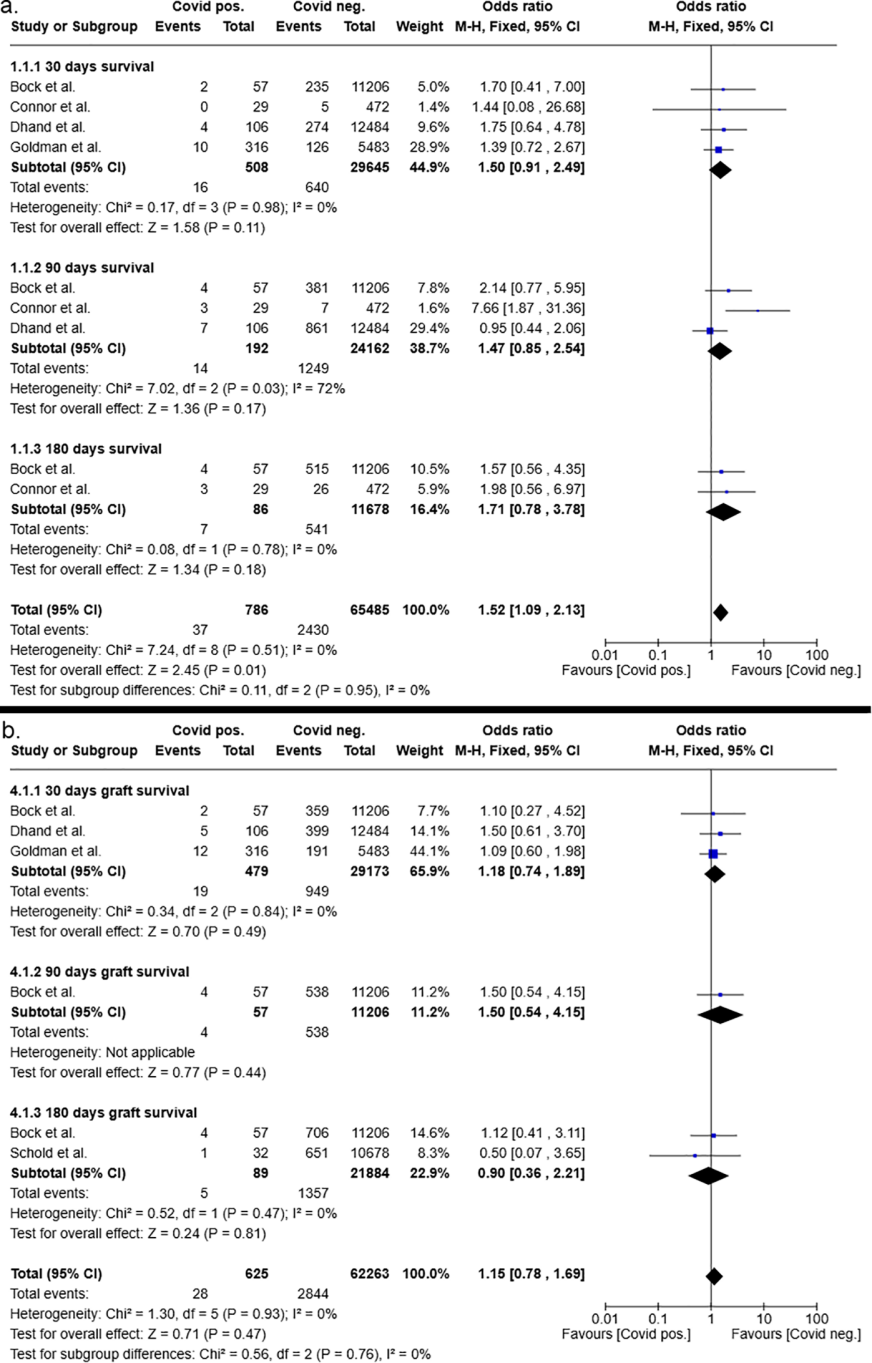
Postoperative 30-, 90- and 180-days patient **(a)** and graft **(b)** survival after liver transplantation from COVID-19 positive and COVID-19 negative donors.

Conversely, the overall patient survival after liver transplantation was significantly improved in recipients of COVID-19 negative grafts (p=0.01) with an overall odds ratio of 1.52 (95%CI 1.09 – 2.13, see **Figure 5**). Subgroup analysis favored COVID-19 negative liver donors, shown by odds ratios of 1.5 (95%CI 0.91 – 2.49, p=0.11) for 30-day patient survival after liver transplantation, of 1.47 (95%CI 0.85 – 2.54, p=0.17) for 90-day patient survival after liver transplantation and of 1.71 (95%CI 0.78 – 3.78, p=0.18) for 180-day patient survival after liver transplantation. The odds ratios at the individual time points did not reach statistical significance.

However, the overall graft survival was not significantly impaired in recipients of COVID-19 positive donors, as determined by odds ratio of 1.15 (95%CI 0.78 – 1.69, p=0.47). Furthermore, subgroup analysis of the 30-,90- and 180-day graft survival after liver transplantation showed no clear trend in favoring COVID-19 negative or COVID-19 positive organs (**Figure 5**).

## 4 Discussion

Since the beginning of the COVID-19 pandemic dynamic changes in the viral infection and its implication for global health care were observed. In consideration of these dynamics, the issue of a recent COVID-19 infection is regarded as a significant topic for our transplantation society. The presented systematic review and meta-analysis are the first to address the impact of a recent COVID-19 infection on liver and kidney transplant patients.

Our analysis determined that a positive COVID-19 donor status did not adversely affect graft survival in liver and kidney transplant recipients compared to patients who received a graft from a COVID-19 negative donor up to 180 days after transplant. In addition, analysis of patient survival outcomes revealed no significant impairment in kidney transplant recipients who received grafts from COVID-19 positive donors in comparison to those recipients with COVID-19 negative donors. In contrast to kidney transplant recipients, liver transplant recipients exhibited a diminished survival rate when the donor had recently tested positive for the virus.

The observed difference in the impact of COVID-19 positive donors on the patient survival of liver and kidney recipients may have been the result of increased recipient-related co-morbidities. In this context, COVID-19 positive livers may have been more frequently allocated to liver transplant recipients in a situation of high urgency (HU). e.g., in acute liver failure. This information, however, was not provided in the studies included. For liver transplant recipients with positive and negative COVID-19 donor status the observed MELD was similar. Nonetheless, a comparison of recipients of a liver or a kidney from a COVID-19 positive or negative donor revealed similar demographic characteristics of the recipients.

It was expected that healthier donors with fewer comorbidities were selected in the COVID-19 positive donor group compared to COVID-19 negative donors. Other than predicted, the comparison of both donor groups showed similar baseline characteristics, including age, BMI, and gender with respect to both liver and kidney transplantation. Indeed, a higher ratio of DCD organs to non-DCD organs and a higher rate of donation after CPR was observed in COVID-19 positive kidney and liver donors (18). Consequently, the underlying results are unlikely to be highly affected by donor-related heterogeneity but might have been influenced by the condition of the recipient, e.g. HU status of liver recipients with COVID-19 positive donors.

In addition, the survival of the graft and recipient may have been affected by the dynamic of the COVID-19 pandemia including the availability of vaccines, antiviral treatment, immune suppression regimes or the variation of pathogenicity of different viral variants (1, 30). It is noteworthy, that the predominant population within this meta-analysis received a vaccination against the virus. Therefore, the pandemic was already advanced at the time of this study. In accordance with the preceding body of literature, there have been no documented cases of viral transmission through kidney or liver transplantation in this investigation.

In contrast to the donor-related studies, the recipient-related studies were more heterogeneous and included only a small number of patients. Thus, any recommendations for the management of postoperative COVID-19 infection in the recipient based on the underlying results are limited. The two included studies indicated that an early COVID-19 infection in recipients was associated with a reduced probability of survival after liver transplantation in comparison to kidney transplantation. The decision to postpone kidney or liver transplantation due to a recent COVID-19 infection in the recipient, however, should remain on a case-by-case basis. There are several factors that contribute to an increased complexity of analyzing recent COVID-19 infection of a recent COVID-19 infection in transplant recipients. Firstly, recipients exhibiting symptoms of a respiratory infection, including elevated inflammation parameters, would be deemed unsuitable for continued transplantation. Furthermore, by the end of 2020, vaccination had become widespread in Europe, and the majority of medical centers ceased to perform transplants for recently infected recipients. In addition, early reports showed an increased mortality rate among transplant recipients who were infected, for example, following a kidney transplant, and among patients undergoing general surgery with recent COVID-19 infection (31, 32). This has led to an increased reluctance to perform kidney or liver transplants on recipients with recent COVID-19 infection.

The authors recommend that the underlying results should be interpreted carefully and do not imply an improving liver or kidney organ quality by a positive COVID-19 status. In light of the reduced number of transplant procedures that occurred during the last years of the COVD-19 pandemic, which resulted in a significant number of patient years lost, it is essential to distinctly delineate the hazardous and beneficial qualities of donors (33). This is particularly relevant given the high mortality rate that has been observed on the waiting list (34). In summary, this investigation provides a comprehensive overview of the impact of early COVID-19 infection on outcomes of liver and kidney transplantation. It is important to note that a positive COVID-19 status in the donor should not be considered an independent risk factor for impaired graft survival. Thus, this analysis may provide insight into future COVID-19 related studies and guidance of the future management of liver and kidney transplant recipients in the event of a viral pandemic.

## Data Availability

The original contributions presented in the study are included in the article/**Supplementary Material**. Further inquiries can be directed to the corresponding author.

## References

[1] El-Shabasy RM Nayel MA Taher MM Abdelmonem R Shoueir KR Kenawy ER . Three waves changes, new variant strains, and vaccination effect against COVID-19 pandemic. Int J Biol macromolecules. (2022) 204:161–8. doi: 10.1016/j.ijbiomac.2022.01.118, PMID: 35074332 PMC8782737

[2] Ponsford MJ Ward TJC Stoneham SM Dallimore CM Sham D Osman K . A systematic review and meta-analysis of inpatient mortality associated with nosocomial and community COVID-19 exposes the vulnerability of immunosuppressed adults. Front Immunol. (2021) 12:744696. doi: 10.3389/fimmu.2021.744696, PMID: 34691049 PMC8526940

[3] WHO . COVID-19 epidemiological update – 16 November 2024 (2024). Available online at: https://www.who.int/emergencies/diseases/novel-coronavirus-2019/situation-reports (Accessed August 30, 2025).

[4] Aubert O Yoo D Zielinski D Cozzi E Cardillo M Dürr M . COVID-19 pandemic and worldwide organ transplantation: a population-based study. Lancet Public Health. (2021) 6:e709–19. doi: 10.1016/S2468-2667(21)00200-0, PMID: 34474014 PMC8460176

[5] Singh P Anand A Rana S Kumar A Prabudh G Kumar S . Impact of COVID-19 vaccination: a global perspective. Front Public Health. (2023) 11:1272961. doi: 10.3389/fpubh.2023.1272961, PMID: 38274537 PMC10808156

[6] Sandoval M Nguyen DT Huang HJ Yi SG Ghobrial RM Gaber AO . COVID-19 mortality may be reduced among fully vaccinated solid organ transplant recipients. PloS One. (2022) 17:e0279222. doi: 10.1371/journal.pone.0279222, PMID: 36542654 PMC9770372

[7] Smith B Nair S Wadei H Mai M Khamash H Schinstock C . Increased mortality in kidney transplant recipients during the delta/omicron era of the COVID-19 pandemic despite widespread vaccination. Clin Transplantation. (2025) 39:e70071. doi: 10.1111/ctr.70071, PMID: 39777936

[8] Knisely A Zhou ZN Wu J Huang Y Holcomb K Melamed A . Perioperative morbidity and mortality of patients with COVID-19 who undergo urgent and emergent surgical procedures. Ann Surg. (2021) 273. doi: 10.1097/SLA.0000000000004420, PMID: 33074900 PMC7737869

[9] Ao G Wang Y Qi X Nasr B Bao M Gao M . The association between severe or death COVID-19 and solid organ transplantation: A systematic review and meta-analysis. Transplant Rev (Orlando Fla.). (2021) 35:100628. doi: 10.1016/j.trre.2021.100628, PMID: 34087553 PMC8137345

[10] Weiss E Dahmani S Bert F Janny S Sommacale D Dondero F . Early-onset pneumonia after liver transplantation: microbiological findings and therapeutic consequences. Liver Transplant Off Publ Am Assoc Study Liver Dis Int Liver Transplant Soc. (2010) 16:1178–85. doi: 10.1002/lt.22132, PMID: 20879016

[11] Gut G Góral A Dal Canton Z Poznański P Krajewska M Kusztal M . Kidney transplantation in COVID pandemic-A review of guidelines. J Clin Med. (2021) 13):10. doi: 10.3390/jcm10132877, PMID: 34209504 PMC8268775

[12] Aasld Expert Panel Consensus Statement . Covid-19 clinical best practice advice for hepatology and liver transplant providers (2024). Available online at: https://www.aasld.org/sites/default/files/2022-10/AASLD%20COVID-19%20Guidance%20Document%2010.06.2022F.pdf (Accessed August 30, 2025). 10.1002/hep.31281 PMC7262242 32298473

[13] TID . Recommendations for Organ Donation and Transplantation after COVID-19 - October 2022 (2024). Available online at: https://tts.org/tid-guidelines/23-tid/tid-news/1255-tid-COVID19-oct2022 (Accessed August 30, 2025).

[14] Carlis R Vella I Incarbone N Centonze L Buscemi V Lauterio A . Impact of the COVID-19 pandemic on liver donation and transplantation: A review of the literature. World J Gastroenterol. (2021) 27:928–38. doi: 10.3748/wjg.v27.i10.928, PMID: 33776364 PMC7968133

[15] Washington Medical School. Donated kidneys from deceased COVID-19 patients are safe to transplant . Available online at: https://medicine.wustl.edu/news/donated-kidneys-from-deceased-COVID-19-patients-are-safe-to-transplant/:~:text=Kidneys%20from%20organ%20donors%20who,Louis (Accessed 10-20-2024).

[16] Sterne JA Hernán MA Reeves BC Savović J Berkman ND Viswanathan M . ROBINS-I: a tool for assessing risk of bias in non-randomised studies of interventions. BMJ. (2016) 355:i4919. doi: 10.1136/bmj.i4919, PMID: 27733354 PMC5062054

[17] McGuinness LA Higgins JPT . Risk-of-bias VISualization (robvis): An R package and Shiny web app for visualizing risk-of-bias assessments. Res Synth Methods. (2021) 12:55–61. doi: 10.1002/jrsm.1411, PMID: 32336025

[18] Bock MJ Vaughn GR Chau P Berumen JA Nigro JJ Ingulli EG . Organ transplantation using COVID-19-positive deceased donors. Am J Transplant. (2022) 22:2203–16. doi: 10.1111/ajt.17145, PMID: 35822320 PMC9349433

[19] Dhand A Okumura K Nabors C Nishida S . Solid organ transplantation from COVID positive donors in the United States: Analysis of United Network for Organ Sharing database. Transpl Infect Dis. (2023) 25:e13925. doi: 10.1111/tid.13925, PMID: 35942924 PMC9538265

[20] Goldman JD Pouch SM Woolley AE Booker SE Jett CT Fox C . Transplant of organs from donors with positive SARS-CoV-2 nucleic acid testing: A report from the organ procurement and transplantation network *ad hoc* disease transmission advisory committee. Transpl Infect Dis. (2023) 25:e14013. doi: 10.1111/tid.14013, PMID: 36694448

[21] Schold JD Koval CE Wee A Eltemamy M Poggio ED . Utilization and outcomes of deceased donor SARS-CoV-2-positive organs for solid organ transplantation in the United States. Am J Transplant. (2022) 22:2217–27. doi: 10.1111/ajt.17126, PMID: 35730252 PMC9350307

[22] Ji M Vinson AJ Chang S-H Merzkani M Lentine KL Caliskan Y . Patterns in use and transplant outcomes among adult recipients of kidneys from deceased donors with COVID-19. JAMA Netw Open. (2023) 6:e2315908. doi: 10.1001/jamanetworkopen.2023.15908, PMID: 37252739 PMC10230314

[23] Romagnoli R Gruttadauria S Tisone G Maria Ettorre G de CL Martini S . Liver transplantation from active COVID-19 donors: A lifesaving opportunity worth grasping? Am J Transplant. (2021) 21:3919–25. doi: 10.1111/ajt.16823, PMID: 34467627 PMC8653300

[24] Montiel Villalonga P Martínez-Alpuente I Fernández-Ruiz M Len Ó Bodro M Los-Arcos I . Transplantation of organs from SARS-CoV-2-positive donors: Preliminary experience from Spain. Transpl Infect Dis. (2023) 25:e14008. doi: 10.1111/tid.14008, PMID: 36659870

[25] Meshram HS Kute VB Patel HV Hegde U Das P Sil K . Is early COVID-19 in kidney transplant recipients concerning enough to halt transplantation? A multicenter comparative analysis from India. Transplant Proc. (2021) 53:2468–75. doi: 10.1016/j.transproceed.2021.08.034, PMID: 34556343 PMC8403672

[26] Moradi A Hadizadeh A Ghiasvand F Ahmadinejad Z Toosi MN Ghazi S . Does COVID-19 infection significantly affect liver transplantation? Results of liver transplantation in the COVID-19 era at a single, high-volume centre. BMJ Open Gastroenterol. (2023) 10. doi: 10.1136/bmjgast-2022-001084, PMID: 36746522 PMC9905753

[27] Connor AA Adelman MW Mobley CM Moaddab M Erhardt AJ Hsu DE . Single-center outcomes after liver transplantation with SARS-coV-2-positive donors: an argument for increased utilization. Transplant Direct. (2024) 10:e1590. doi: 10.1097/TXD.0000000000001590, PMID: 38464428 PMC10923316

[28] Koval CE Eltemamy M Poggio ED Schold JD Wee AC . Comparative outcomes for over 100 deceased donor kidney transplants from SARS-CoV-2 positive donors: A single-center experience. Am J Transplant. (2022) 22:2903–11. doi: 10.1111/ajt.17203, PMID: 36176236 PMC9538585

[29] Yamauchi J Azhar A Hall IE Bhalla A Potluri VS Tanriover B . Comparison of short-term outcomes in kidney transplant recipients from SARS-coV-2-infected versus noninfected deceased donors. Clin J Am Soc Nephrol. (2023) 18:1466–75. doi: 10.2215/CJN.0000000000000275, PMID: 37574663 PMC10637460

[30] Delft Av Hall MD Kwong AD Purcell LA Saikatendu KS Schmitz U . Accelerating antiviral drug discovery: lessons from COVID-19. Nature reviews. Drug Discov. (2023) 22:585–603. doi: 10.1038/s41573-023-00692-8, PMID: 37173515 PMC10176316

[31] Nepogodiev D Bhangu A Glasbey JC Li E Omar OM Simoes JFF . Mortality and pulmonary complications in patients undergoing surgery with perioperative SARS-CoV-2 infection: an international cohort study. Lancet. (2020) 396:27–38. doi: 10.1016/S0140-6736(20)31182-X, PMID: 32479829 PMC7259900

[32] Udomkarnjananun S Kerr SJ Townamchai N Susantitaphong P Tulvatana W Praditpornsilpa K . Mortality risk factors of COVID-19 infection in kidney transplantation recipients: a systematic review and meta-analysis of cohorts and clinical registries. Sci Rep. (2021) 11:20073. doi: 10.1038/s41598-021-99713-y, PMID: 34625642 PMC8501014

[33] Martinez-Reviejo R Tejada S Cipriano A Karakoc HN Manuel O Rello J . Solid organ transplantation from donors with recent or current SARS-CoV-2 infection: A systematic review. Anaesthesia Crit Care Pain Med. (2022) 41:101098. doi: 10.1016/j.accpm.2022.101098, PMID: 35533977 PMC9074299

[34] Menon G Li Y Musunuru A Zeiser LB Massie AB Segev DL . COVID-19 and access to kidney transplantation for older candidates in the United States: A national registry study. Kidney Med. (2024) 6:100756. doi: 10.1016/j.xkme.2023.100756, PMID: 38205431 PMC10777077

