# Supplementary material for: Impact of recent COVID-19 infection on liver and kidney transplantation – a worldwide meta-analysis and systematic review
Source: Front Immunol. 2025 Sep 29;16:1626391. doi: 10.3389/fimmu.2025.1626391 (PMC12515668; doi:10.3389/fimmu.2025.1626391)
Supplement: Supplementary file 1 [file Table1.docx]

**Supplementary material:**

Table 1) Search strategy

| Nr. | Terms | |
| --- | --- | --- |
| 1. | COVID-19/ | |
| 2. | Coronavirus Infections | |
| 4. | (sars-cov-2 or sars-cov2 or sarscov2 or sarscov-2). | |
| 6. | coronavirus. | |
| 7. | kidney transplantation/ | |
| 8. | ((kidney or renal) adj1 (transplant* or graft* or allograft*)). | |
| 9. | liver transplantation/ adj1 (hepatic(transplant* or graft* or allograft*)) | |
| 10. | ((liver or hepatic) adj1 (transplant* or graft* or allograft*)) | |
| 15. | or/16-17 | |
| Database | MeSH terms | |
| Cochrane library/ Europe PMC | (liver OR hepatic OR kidney OR renal) AND (transplantation* OR transplant* OR graft*) AND (COVID OR Sars-Cov2 OR coronavirus) AND (donor OR recipient) | |
| PubMed | (Liver transplantation) OR (Hepatic transplantation) OR (kidney transplantation) OR (Renal transplantation) AND (COVID OR Sars-Cov2 OR coronavirus)) AND (adult) AND (donor) OR (recipient) | |
| WHO COVID-19 database | (Kidney OR liver) AND (Covid 19 positive donor OR Covid 19 positive recipient) AND (adult) AND (transplantation) | |
| PiCo Search |  | |
| Population | Intervention | Outcome |
| Adult | Covid-19 RT-PCR testing | Survival |
| Liver Transplant |  | Overall survival |
| Kidney Transplant |  | Graft rejection |
| COVID-19 |  | Death |
